# Investigating potential supply of ecosystem services in cultural landscapes through efficiency analysis

**DOI:** 10.1007/s00267-024-01967-5

**Published:** 2024-04-11

**Authors:** Vasja Leban, Lidija Zadnik Stirn, Špela Pezdevšek Malovrh

**Affiliations:** University of Ljubljana, Biotechnical Faculty, Department of Forestry and Renewable Forest Resources, Večna pot 83, SI-1000, Ljubljana, Slovenia

**Keywords:** Ecosystem services, Multifunctionality, Data envelopment analysis, Tobit regression, Slovenia

## Abstract

One of the paramount challenges in natural resource management revolves around the delicate equilibrium between the demand for and the supply of diverse Ecosystem Services (ESs) within a cultural landscape. Recognizing the centrality of cultural landscapes to human well-being, the sustainability of these landscapes hinges upon the health and stability of ecosystems that can effectively provide the required ESs. Over the long term, the sustainable supply of ESs is constrained by the potential supply of ESs. Understanding the potential supply of ESs is crucial for averting compromises to the ecosystems within a landscape. This article introduces a novel perspective on evaluating the ESs of a landscape by means of efficiency analysis. Instead of presenting the potential supply of ESs in absolute terms, we offer a comparative analysis of ESs' relative supply to associated management costs. In principle, the efficiency of Landscape Units (LUs) is defined as the ratio of the potential supply of multiple ESs to the costs associated with land use and land cover management. The resultant efficiency maps serve as hot and cold spot maps, revealing efficient ecosystem compositions that yield multiple ESs. This composition reflects management efforts, incorporating various management costs. Forests emerge as pivotal ecosystems in landscapes, delivering the most ESs at the lowest costs. These efficiency maps offer valuable insights for regional planners, enabling them to enhance the supply of ES in inefficient LUs by studying the ecosystem structure and associated costs of the most efficient LUs.

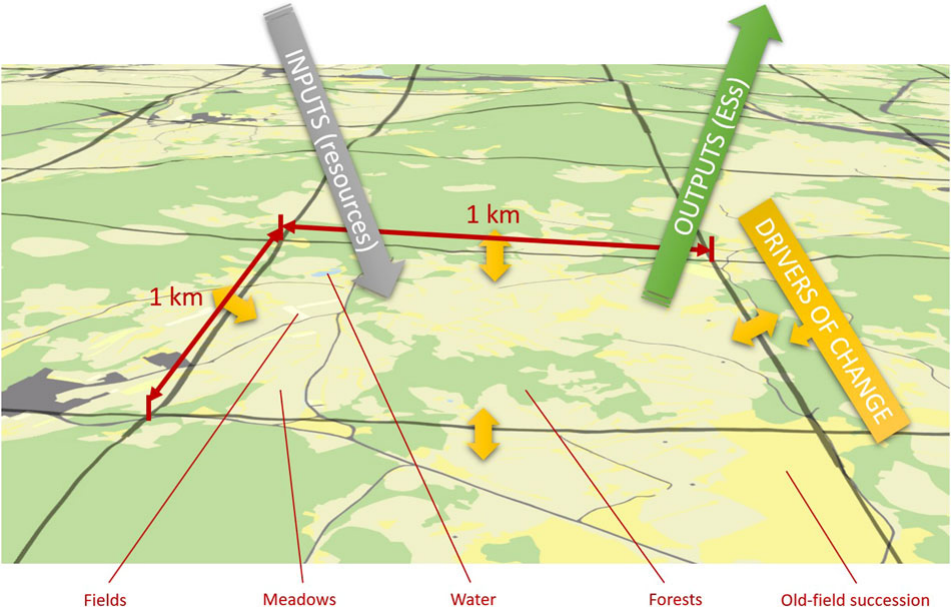

## Introduction

The potential supply of multiple ESs that can be provided in a sustainable manner depends on the composition of land use and land cover (LULC) and ecosystem conditions of that specific landscape (Burkhard and Maes 2017, pp. 153–154). This potential supply is site-specific and dynamic due to natural change and other drivers of change (e.g., socioeconomic, political, and technological; ibid.). Assessing ES supply on a landscape level is essential since it is a function of ecosystems and reflects the various landscape characteristics, such as mosaics, the presence of certain landscape features, and natural phenomena, alongside other factors (e.g., geomorphological, political). Not all areas in the landscape supply all ESs at the same time or in equal amounts, and not all ESs are demanded constantly (e.g., throughout the year) in certain places. In addition, various ecosystem interactions may exist that either enhance or reduce certain ESs supplies (Raudsepp-Hearne et al. 2010; Turner et al. 2014). As Raudsepp-Hearne et al. (2010) pointed out, one of the most important and puzzling questions is how to manage multiple ES in different landscapes.

Over the past decade, several research efforts that present novel approaches to address this issue have been conducted. These include an ESs landscape index to compare the multifunctionality of landscapes in different communities (Rodríguez-Loinaz et al. 2015), a Multiple ESs Landscape Index (MESLI) calculated as the sum of normalized values of selected ES indicators (Shen et al. 2020), and an Ecosystem Service Bundle Index (ESBI) using structural equation modeling (Hong et al. 2020). Shaad et al. (2022) developed an indicator framework and a Fresh-Water Health Index to assess both the supply of and the demand for water ES. Vargas et al. (2019) emphasized the role of accounting frameworks in supporting decision-making and developed a remote sensing method to quantify ES supply and ecosystem capacity to provide ESs. Previously, Nelson et al. (2009) introduced a spatial modeling tool InVEST, to assess and map multiple ESs at the landscape level. Their approach uses a combination of scenario analysis and LULC maps (along with other relevant parameters) to create ESs maps and evaluate trade-offs. The importance of landscape-level multifunctionality has also been recognized by Fagerholm et al. (2019), who undertook a Europe-wide survey to assess the perceived benefits of ESs by local communities.

While these studies understand ESs as “outputs” of ecosystems in a landscape, not many studies acknowledge the various intakes or “inputs” to ecosystems that are required to provide a given set of ESs. Trabucchi et al. (2014) assessed and mapped ES at a watershed level and proposed to account for ESs with degradation maps. Susaeta et al. (2016) used the Data Envelopment Analysis (DEA) method to evaluate the efficiency of supplying different ESs in forests. Kapfer et al. (2012) evaluated the contribution of agricultural land to environmental services and landscape benefits using DEA with a combination of statistical methods. Ibrahim et al. (2021) used a similar two-step approach to assess the efficiency of socio-ecological systems and factors at the international scale. Furthermore, Favretto et al. (2016), Wam et al. (2016), and Schwenk et al. (2012) employed a multi-criteria decision-making (MCDM) approach to assess multiple ESs or evaluate (opportunity) costs of multiple ESs use. In principle, several alternative ESs under specific conditions have been assessed in terms of costs and trade-offs in relation to the supply of ESs. Another framework to identify and prioritize areas requiring active restoration in urban ecosystems using MCDM) methods was presented by Mostert et al. (2018). Marttunen et al. (2022) reviewed 23 articles that combined the use of ESs concept and MCDM methods in water management and concluded that combining the ES concept with MCDM provides a more transparent and objective multi-stakeholder process. In particular, MCDM methods are extremely valuable for the process of weighting, which tends to be cognitively demanding if the questions and guidance are inadequate. Finally, Uhde et al. (2015) explored the use of MCDM in forest management planning and specifically addressed stakeholder engagement, and the trade-offs between ESs and uncertainty.

In this paper, we advance the state of the art by presenting an approach for identifying and analyzing the potential supply of multiple ESs at the landscape level as a function of specific inputs. Further developing the results of Vejre et al. (2015), we propose that LULC composition and the associated management costs are good input indicators of the potential supply of ESs at the landscape level. In this approach it is of crucial importance that we are aware that these costs might not adequately reflect the actual costs of providing various ESs. The management costs in our study are costs that arise for management for specific LULC, which means that no other costs (e.g., infrastructural, cultural) were considered, nor were the paybacks from e.g., subsidies, municipality aids, or other (social) transfers. Moreover, the opportunity costs of supplying one or some ESs at the expense of others are also not included. Porto et al. (2014) concluded that despite the multiplicity of landowners and interests, a landscape can still provide objectives or, in other words, ESs and benefits to multiple stakeholders. The aim of this paper is to present the developed conceptual framework and proposed methodology for analyzing the potential supply of multiple ESs at the landscape level. Three research objectives were set:

- To map the potential supply of 17 ESs in the study area.
- To conduct efficient analysis of landscapes in the study area.
- To assess the fundamental drivers of change that affect landscape efficiency.

## Conceptual framework

Previous studies have shown that human well-being is derived from ESs defined by functioning, healthy, and biodiverse ecosystems (e.g., Kumar 2010; Villamagna et al. 2013). ESs supply and biodiversity depend on ecosystem ecological structures and processes, which are influenced by direct and indirect (external) drivers of change, such as land conversion and habitat change, demographic, economic, and political. To some extent, these ecosystems are the result of policy-level decision-making and operational-level management. Decision-making at the policy level requires the management of relationships among all stakeholders with the ultimate goal of finding a satisfactory solution to the spatial and temporal composition of LULC at the landscape level. Management, on the other hand, requires direct intervention on the ground by landowners and land managers. Landscape-level analysis is appropriate for both regional planning and ESs assessment. Ecosystems are defined in this study as natural and semi-natural ecosystems only, excluding artificial ecosystems such as urban ecosystems. Examples of ecosystems considered in the study include forests, pastures, fields, old field succession, and water.

**Fig. 1:**
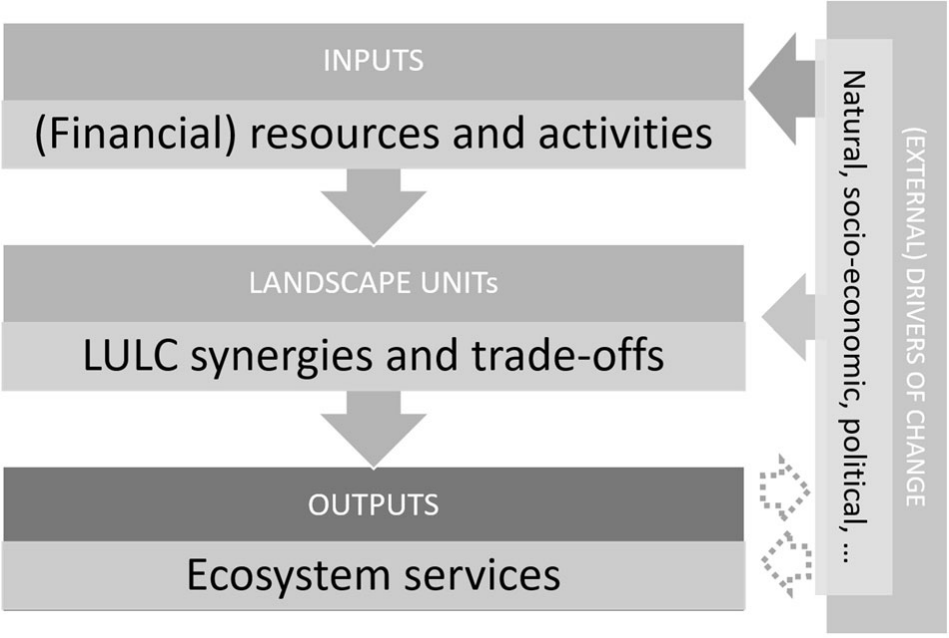
Conceptual framework adopted in the study (own elaboration)

To avoid ambiguity, we use the name landscape units (LUs) for the remainder of this article to denote the basic spatial unit used in this study (cf. Rolo et al. 2021). In this study, LUs are the analytical unit and are defined as a square grid cell with an arbitrarily defined side length. LUs are *service-providing units* (SPU) and service-benefiting units (SBA) simultaneously since they supply ESs and at the same time, the benefits are delivered within the same spatial unit (i.e., in situ SPU–SBA relation as defined by Burkhard et al. 2014). LU are small landscape areas with a given LULC composition (i.e., composed of natural and semi-natural ecosystems) with the potential to provide certain ESs based on their unique ecosystem features and societal demand. Each of these LUs can be further described with characteristics such as the predominant bedrock, average slope, LULC composition, presence/absence of nature protection areas and others. Importantly, to deliver ESs, ecosystems require proper management of resources, activities, materials, and labor (Burkhard et al. 2014). Management is provided to each LULC and can be expressed as the cost of maintaining the given LULC, from which various ESs may arise. Therefore, in this study, we consider these management resources and activities as the “inputs” that allow the supply of the “outputs”, that is ESs. Consequently, we can analyze the relationship between inputs and outputs in each LU and compare the results among LUs, as in benchmarking or efficiency analysis. In principle, the efficiency of a LU in terms of human benefits increases as the number of outputs it produces with the fewest inputs increases. Finally, after completing the efficiency analysis, the external drivers that affect this efficiency can be assessed. This can be done, for example, through regression analysis. The graphical representation of the conceptual framework is shown in Fig. 1 and is summarized in the graphical abstract.

To operationalize the described framework, we continue the article with the following proposition: the higher the ratio between the potential supply of multiple ESs and the resources needed to manage the LULC composition of the given LU, the better this LU is compared to other LUs. An LU is considered (Pareto) efficient when it cannot improve the supply of ESs with given inputs without making another LU worse off. Efficient LUs are benchmark units that indicate appropriate resource management. The “efficiency approach” was chosen over other potential alternatives such as machine learning, stochastic methods, and Bayesian frameworks. This approach was selected because it offers an intuitive pathway and allows for effective consideration of multiple, scale-dependent, and non-normally distributed parameters. While other approaches may offer more nuanced results, they may require the variables to be normally distributed, additional modifications of the original data, or the use of fewer parameters, the efficiency approach was considered the best fit for this particular situation.

The results of this study could be therefore used at the regional level by the sectoral authorities (e.g., forestry, agriculture, nature protection) to aid their communication and help design better policy measures. The methodology developed in this research is based on the following premises:

- a natural and semi-natural LULC composition in each LU has been achieved by decisions of multiple stakeholders; this combination can be considered a “consequence” of stakeholders’ decisions to invest or not invest management resources;
- each LULCs composition in LU inherently provides a certain combination of ESs; in principle, LULCs have the potential to supply all relevant ESs, but only a few ESs are realized within a certain LU at any given time;
- the potential supply of ESs in an LU can be “weighted” by the average costs for managing each LULC in the LU; these costs can be considered as proxies for supplying that specific ESs combination within the LU.

## Methods

### Study area

**Fig. 2:**
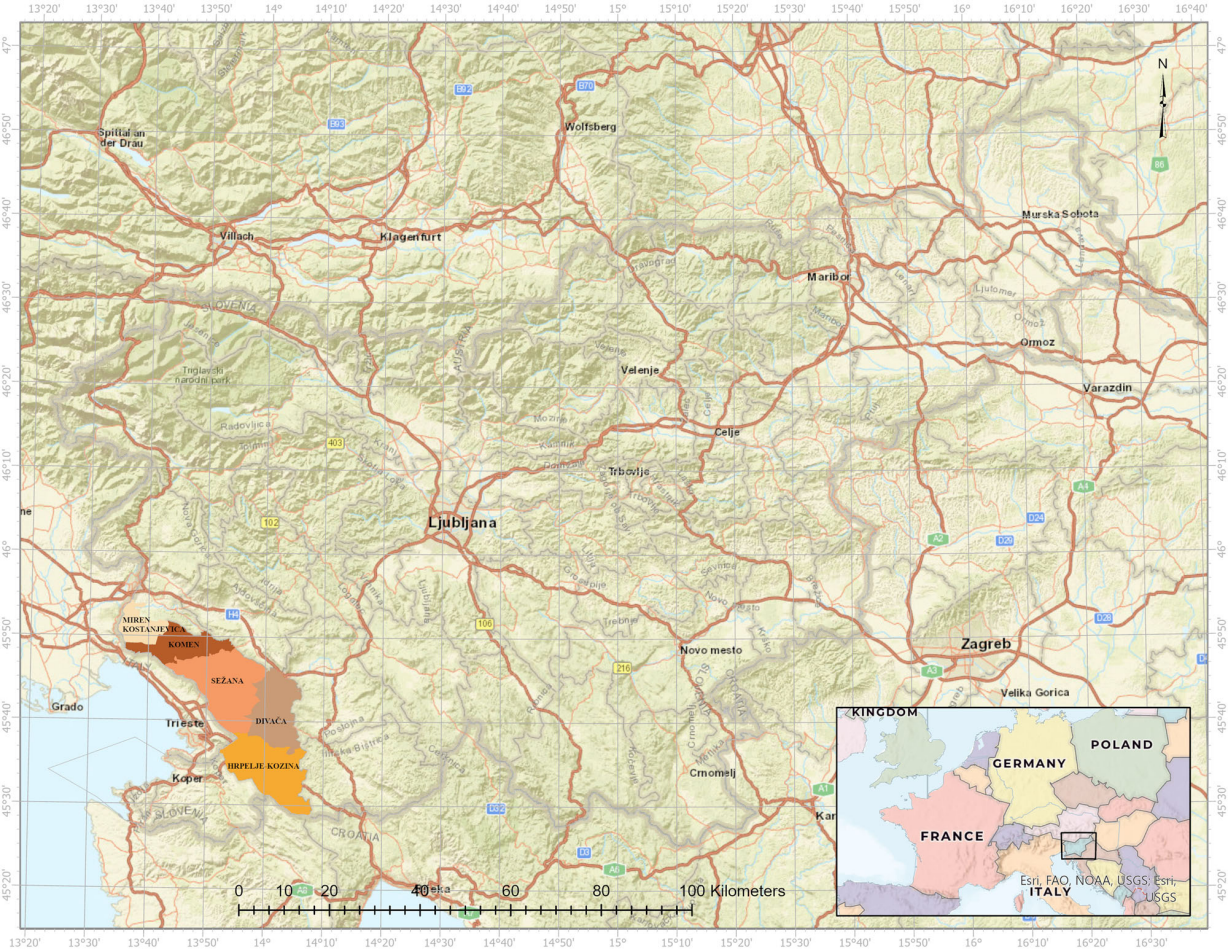
Study area represented by the five municipalities

To substantiate the proposed framework, the empirical part of this study was conducted in southwestern Slovenia, a region with a sub-Mediterranean climate that covers approximately 72,288 hectares and is home to about 30,000 inhabitants. It spans the Karst, Brkini, and Sub-Dinaric Littoral regions and includes five municipalities, namely Sežana, Komen, Divača, Hrpelje-Kozina, and Miren-Kostanjevica (Fig. 2). The economic development of the region was based on agriculture until the second half of the 19th century, followed by viticulture and stonemasonry after the railroad was built (Panjek 2015). After World War II, the level of education increased, and at the same time, the industrial sector developed, leaving traditional landscape features such as dry-stone walls, tiny shepherds’ houses, stone wells, and old fields, and succession began. People abandoned agriculture in search of better and easier incomes in the emerging automotive, timber, and logistics industries or other industries in larger cities such as Koper and Sežana. This transition also led to migration to larger cities, changing people’s lives and reorienting their needs and resources to other areas of activity.

The Natura 2000 network covers 70% of the study area, which is also considered one of the European biodiversity hotspots (Živi Kras-Carso 2014). The study area is in a hilly topography at an altitude between 200 and 800 m above sea level (Marušič et al. 1998). Forests cover 65% of the area, followed by meadows and pastures (20%) and old field succession (4%). Agricultural land occupies less than 3% of the area, urban and artificial land about 4% in traditionally scattered settlements. There is only less than 0.1% surface water, mainly due to the predominantly calcareous bedrock. Water is found mainly in underground caves, passages, and natural pools.

Traditionally, the study area was mainly used by the local residents for agricultural purposes, especially as pastureland. In the past, the forests were sparse and consisted mainly of deciduous species such as oaks, hornbeams, and ash trees. In fact, the grasslands that dominated the Karst (most of the northerly part of the study area) at the end of the 18th century were transformed mainly into forests over the next 250 years (Kaligarič and Ivajnšič 2014). This transition was made possible by the artificial introduction of a non-native Austrian pine (*Pinus nigra* Arnold) in the 19th century. This introduction, which was considered positive and beneficial by foresters and policymakers, led to prolonged resentment among local residents and landowners, who were deprived of their livelihoods as a result (Remec 2021). Eventually, local residents accepted the new species, which became an integral part of their identity. Nowadays, the Austrian pine is losing vitality, and more native species (e.g., *Quercus petraea* (Matt.) Liebl., *Ostrya carpinifolia* Scop.) are shaping the structure of forest undergrowth. The study area thus represents a traditional cultural landscape that is now returning to its natural state, making it an interesting subject for research. And since similar cultural landscapes can be found all over the world, we believe it is a suitable study region.

### Mapping and assessing the potential supply of ESs

Based on the preliminary research conducted in the study area and after reviewing the existing literature (e.g., Panjek 2015; Marušič et al. 1998; Živi Kras-Carso 2014), seventeen ESs were selected from three main ES groups, namely regulating, cultural, and provisioning. The preliminary research encompasses face-to-face interviews with stakeholders in the study area prior to the ES mapping and assessment. The nineteen stakeholders interviewed were local residents, farmers, foresters, hunters, agricultural advisory officers, a municipal officer, a tourist guide, a cultural worker, and a student. The interviewees were asked, among other things, which ESs are considered important for most people in the study area. After analyzing the results, a list of identified ESs was created. The naming of ESs follows the CICES v5.1 classification on a group-, class- or class-type level. Table 1 shows the selected ESs, ES groups, short names, indicators, data sources, and method of mapping.

**Table 1:** List of evaluated potential supplies of ESs, indicators used, data sources, and methods

Ecosystem service	ES short name	ES group	Indicator [unit]	Data source	Method/Model
Control of erosion rates	Erosion control	Regulating	Presence of erosion control measures [-]	Slovenian Meteorological Agency	GIS overlay
Regulation of temperature and humidity, including ventilation and transpiration	Climate regulation		Carbon stored in soil carbon pool [Mg C km^−2^]	Skudnik et al. 2021; Šinkovec et al. 2021; Buosi et al. 2021	GIS overlay
Characteristics of living systems that enable education and training	Educational	Cultural	Presence of protected areas [n km^−2^]	Ministry of Environment and Spatial Planning	GIS overlay
Physical and experiential interactions with natural environment - touristic	Touristic		Average photographs per user day from 2005–2017 [-]	Flickr through InVEST	InVEST Visitation Recreation and Tourism
Experiential interactions with natural environment - flora observation	Flora observation		Number of different tree species [n km^−2^]	Slovenia Forest Service	Excel calculations, GIS overlay
Experiential interactions with natural environment - fauna observation	Fauna observation		Presence of protected wild animals [n]	Kaczensky et al. 2021	GIS overlay
Characteristics of living systems that are resonant in terms of culture or heritage	Inspirational		Number of immobile cultural heritage sights [n km^−2^]	Ministry of Culture	GIS overlay
Characteristics of living systems that that enable activities promoting recuperation or enjoyment through active interactions - hunting	Hunting		Number of different wild animals [n km^−2^]	Slovenia Forest Service	GIS overlay
Elements of living systems that have sacred or religious meaning	Spiritual		Number of intangible cultural heritage sites [n km^−2^]	Ministry of Culture	GIS overlay
Physical and experiential interactions with natural environment	Recreation		Recreation potential index [-]	Ministry of the Environment and Spatial Planning; EEA, 2022; Ministry of Agriculture, Forestry and Food	Excel calculations, GIS overlay
Characteristics of living systems that enable esthetic experiences	Esthetic		Landscape visibility (given obstacles) [-]	Ministry of Environment and Spatial Planning	InVEST Scenic Quality
Cultivated terrestrial plants	Crops	Provisioning	Soil index [-]	Ministry of Agriculture, Forestry and Food	GIS overlay
Medicinal wild plants for nutrition	Medicinal plants		Number of medicinal plants gathering households [n km^−2^]	Slovenian Statistical Survey	Excel calculations, GIS overlay
Ground water for drinking	Water		Distance to the nearest water spring or water body [m]	Ministry of Agriculture, Forestry and Food	GIS overlay
Wild plant used for nutrition	Wild plants		Volume of chestnut in forest growing stock [m^3^ ha^−1^]	Slovenia Forest Service	Excel calculations, GIS overlay
Wild animal used for nutrition	Wild animals		Number of hunted animals [n ha^−1^]	Slovenia Forest Service	Excel calculations, GIS overlay
Wild trees for materials or energy	Timber production		Forest growing stock [m^3^ ha^−1^]	Slovenia Forest Service	GIS overlay

Based on the conceptual framework described above, square LUs (i.e., rectangular cells) with a side length of 1 km were chosen. The grid surrounding the study area was built using the *Create Fishnet* tool in ArcGIS PRO 3.1 (ESRI) software. LUs that were completely outside of the study area were excluded, as well as LUs with an area less than 0.999 km^2^. These LUs were excluded to ensure comparability between LUs and avoid issues related to their size. The grid’s final composition consists of 627 cells. We used detailed LULC maps of the Slovenian Ministry of Agriculture, Forestry, and Food as a basis for assessing the potential supply of ESs and for visualizing the results. We employed a deterministic approach to map the potential supply of ESs through direct or indirect measurements, mainly using proxy indicators. The usage of LULC-based proxy indicators is considered as not preferred alternative, especially for small-scale analyses (Eigenbrod et al. 2010). On the other hand, when information on ecosystem conditions and direct supply of ESs are inexistent or insufficient, proxies are necessary to develop (Burkhard and Maes 2017).

Nevertheless, two ESs (i.e., landscape esthetics and tourism) were assessed by modeling with a modeling tool, and five ESs (i.e., climate regulation, flora observation, crop production, wild plant food, wood production) were assessed directly with the LULC layer and parameters provided by the authorities. The list of ESs, indicators, data sources, and methods is shown in Table 1. It is of utter importance to note that we opted for indicators that were spatially explicit and publicly available. For this reason, some indicators do not entirely match with the selected ESs name (e.g., flora observation, fauna observation). The reported mapped ESs were rescaled using min-max normalization between 0 and 100 to account for the different scales. This involved subtracting the minimum values from the dataset values and dividing the intermediate result by the difference between the maximum and minimum values of the dataset (Rolo et al. 2021). The final step was to multiply the result by 100 to obtain a range between 0 and 100. As noted by Paracchini and Capitani (2011), building composite indicators requires non-dimensional variables. In our study, we compare the weighted ES values with their corresponding costs using a complex procedure rather than simply adding up values. For technical reasons related to the limitation of the DEA software used (i.e., zero values could lead to infinite cycling of the software, yielding no results), we replaced zero values of inputs and outputs with the value 0.001 (Coelli et al. 2005).

*Erosion control* was mapped using the presence of erosion control measures published by the Slovenian Meteorological Agency. We assign values to LUs that are proportional to the area covered by these measures, assuming that ecosystems present in LUs with erosion control measures provide lower erosion control potential (Crossman et al. 2013). ES *climate regulation* was mapped as a proxy value of carbon stored in soils (Burkhard and Maes 2017; Wei et al. 2017). Each LULC was assigned a corresponding carbon storage value, and then a weighted arithmetic mean was calculated for each LU. Carbon values for LULCs with sources and calculation methods are listed in Table A.1 (see Appendices).

*Educational* ES has been mapped by the presence of protected natural areas (e.g., Natura 2000 sites, ecologically important areas). The larger the share of protected areas in a LU, the higher the potential supply of ESs on that LU, as these areas are important to promote environmental awareness through education (De Dominicis et al. 2017). *Touristic* ES has been modeled using the InVEST model Visitation Tourism (Baró et al. 2016, Outdoor Recreation Activities—Supply 2018, Sharp et al. 2021). The InVEST Visitation tourism model displays average visitation rates in different landscapes for the period based on Flickr photos with geotags that were taken in a specific area. Since we wanted to obtain a long-term average, we chose to use the maximum period, i.e., 2005–2017. ES *flora observation* was mapped as the diversity of tree species—the higher the number of different tree species in the forest, the higher the potential to provide this ES – aggregated for LUs. A similar indicator has been used for assessing biodiversity appreciation (e.g., Queiroz et al. 2015). *Fauna observation* ES was mapped by the presence of protected large carnivores (i.e., brown bear, lynx, and wolf). The larger the share of wildlife territories present in a LU, the higher the potential supply of ESs on that LU, as the animals can be seen in that area. Species richness (n ha^−1^) is often used as an indicator (e.g., Crouzat et al. 2015), but due to a lack of data, we used the abundance of the territory per square kilometer. *Inspirational* ESs have been mapped based on the density of immobile cultural heritage sites. The idea is that a higher potential for providing ES lies in areas with a higher density of cultural heritage sites. Immobile cultural heritage sites (e.g., buildings and monuments) serve as inspiration for culture, art, and design (Berglihn and Gómez-Baggethun 2021). Spatial explicit maps with sites’ locations were appended to each LU to obtain the number of locations. The number of different wild animals that can be hunted found in the LU was used as a proxy for ES *hunting*. This indicates the distribution of wild animals and the diversity of hunting experience, as more species offer more opportunities for various hunters. Importantly, in this study, we consider ES hunting as a cultural ES and ES wild animals used for nutrition as a provisioning ES (Turner et al. 2014; Schulp et al. 2014). *Spiritual* ESs have been mapped based on the density of immaterial cultural heritage sites. Higher potential for providing ES lies in areas with a higher density of intangible cultural heritage, e.g., performing arts, traditional knowledge, practices, and rituals, which provide a foundation for spiritual experiences in nature (Berglihn and Gómez-Baggethun 2021). *Recreational* ES was evaluated using the recreational potential index, which has been described in detail by Paracchini et al. (2014) and Outdoor Recreation Activities—Supply (2018). The recreation potential index is calculated as the average normalized value of six indicators, namely recreation value of protected areas, degree of naturalness, distance to water bodies, number of LULC types, terrain roughness, and density of mountain peaks. Due to the low density of significant mountain peaks in the area, this indicator was replaced by the distance from the centroid of each LU to the nearest of the 48 mountain peaks. The only criterion for selecting the peaks was an elevation of more than 250 m above sea level. *Esthetics* ESs have been modeled using the InVEST model Scenic Quality (Baró et al. 2016, Outdoor Recreation Activities—Supply 2018, Sharp et al. 2021). For the InVEST Scenic Quality model, a digital elevation model and data from the Ministry of the Environment and Spatial Planning were used to identify features that negatively impact scenic quality (e.g., surface mines and industrial areas). The areas with unaffected views were considered as the areas that potentially supply the most ES landscape esthetic.

The soil index calculated by the Ministry of Agriculture, Forestry, and Food was used as a proxy for ES *crops*. The soil index can be interpreted as a “productivity index” as it indicates the productive capacity of the soil (Egoh Benis et al. 2012). The higher the values, the more productive the soil is and thus more suitable for agricultural production. The number of households collecting medicinal plants was used as a proxy for ES *medicinal plants*. We used Lovrić et al. (2020) estimate that 37.9% of Slovenian households collect wild medicinal and aromatic plants and calculated the number of households per settlement—the higher the number, the higher the potential supply of ES. Importantly, this indicator does not reflect the actual availability of certain plants in the ecosystems near the household. However, it is assumed that individuals are familiar with the flora in their immediate vicinity and therefore gather plants in close proximity to their residences. *Water* was mapped by calculating the nearest distance to an above-ground water body or registered water source. Proximity to water bodies is a proxy indicator of water yield; the closer the water body, the higher the potential supply of water (cf. Burkhard and Maes 2017; Vigerstol and Aukema 2011). The volume of chestnut in the forest stand was used as a proxy for assessing ES *wild plants* (Burkhard et al. 2014). Chestnut is the only species used as a proxy for plants in general, as no other flora (e.g., mushrooms) has been mapped or spatially assessed previously. The number of wild animals hunted was used as a proxy for ES *wild animals*. We used the Slovenia Forest Service data on a number of shot wild animals that are often used for nutrition per hunting district (Burkhard et al. 2014; Raudsepp-Hearne et al. 2010). Finally, forest growing stock in the forest stand was used as a proxy for assessing ES *timber production* (Burkhard et al. 2014). This indicator is related to forest areas only and shows the capacity of a forest stand to produce wood.

### Efficiency of landscape units

DEA was used to evaluate the efficiency of landscape units in supplying various ESs (i.e., outputs), given management costs (i.e., inputs). DEA is a popular nonparametric method for measuring decision-making unit (DMU) efficiency developed by Charnes et al. (1978). In this study, the concept of DMUs is equivalent to the concept of LUs. DEA allows the use of multiple inputs and multiple outputs with different units. This means that one can assess multiple economic, environmental, and social aspects without having to deal with units and data transformations (Kapfer et al. 2012). The more outputs produced with given inputs, the more efficient the DMU (note: in our study, we use LU as equivalent to DMU). DEA uses linear programming to calculate efficiency as the ratio between the weighted sum of outputs and the weighted sum of inputs and can be represented as described below (Coelli et al. 2005, Huguenin 2013). Suppose we have *j* different *LU*_*j*_*, j* = *1,…, n* and each *LU*_*j*_ requires *m* inputs *I, i* = *1,…, m*, in order to produce *s* different outputs *r, r* = *1,…, s*. A *LU*_*j*_ requires *x*_*ij*_ units of input *i* to produce *y*_*rj*_ units of output *r*. We assume that each *LU*_*j*_ has at least one positive input and one positive output, where *x*_*ij*_ and *y*_*rj*_ are bigger or equal to 0. We want the technical efficiency *LU*_*j*_ to be maximized (Eq. 1) as to other *LU*_*j*_, *j* = *1, …, n*. The difference between weighted outputs and weighted inputs is greater than or equal to zero for all *LU*_*j*_ (Eq. 2). The maximum technical efficiency value of each *LU*_*j*_ is 1 (Eq. 3), and the values of weights *u*_*r*_ and *v*_*i*_ must be greater than 0 (Eq. 4).

1 $${\rm{maximize}}\mathop{\sum }\limits_{r=1}^{s}{u}_{r}{y}_{{rk}}$$

subject to

2 $$\mathop{\sum }\limits_{i=1}^{m}{v}_{i}{x}_{{ij}}-\mathop{\sum }\limits_{r=1}^{s}{u}_{r}{y}_{{rj}}\ge 0,j=1,\ldots ,n,$$

3 $$\mathop{\sum }\limits_{i=1}^{m}{v}_{i}{x}_{{ik}}=1,$$

4 $$\begin{array}{ccc}{u}_{r},{v}_{i} \,>\, 0 & \forall r=1,\ldots ,s{\rm{;}} & i=1,\ldots ,m.\end{array}$$

In our study, we are interested in identifying the LUs with the highest potential supply of multiple ESs, given the estimated cost of management per LULC, so we opted for *output orientation*. Since the LUs have the same acreage and we assume they are operating at optimal scale, *constant returns to scale* technology were applied. The 17 ESs evaluated were used as outputs in the efficiency analysis. We calculated one input, i.e., the cost of managing the various LULCs within an LU, which is a proxy for obtaining the set of ESs in that specific LU (Table A.2 in the Appendices). Specifically, the associated management costs from the economic model calculations of the Agricultural Institute of Slovenia and the Slovenian Forestry Institute were assigned to the respective LULCs and averaged within each LU. Urban and artificial areas were excluded from the average cost calculations and were not included in the total area of LUs because they do not represent a natural or semi-natural ecosystem and, thus, do not provide ES for the purposes of this study.

The idea behind assigning the average management cost of producing products associated with a particular LULC composition to LUs as a proxy lies in the fact that the supply of various ESs is theoretically the result of independent LULC management for a particular use. Take pasture, for example: if a landowner manages it for sheep herding, that pasture is also a (potential) storage area for carbon in the soils, an area for observing and studying flora and fauna, a landscape of inspiration, and other benefits. The cost of “co-producing” all these ESs is therefore proportional to the management cost of producing the main products or services (e.g., grazing in the example from which dairy or meat products are produced). This is only true for a static temporal analysis with ESs that are strongly connected with particular LULCs. In a dynamic configuration and with other indicators, this assumption may not hold. In other words, the use of pastureland also reflects the potential contribution of that pastureland to the supply of various other ESs, including those indirectly associated with a particular ecosystem. *Win4Deap v2.1* (^©^ 2015 Michel Deslierres) was used for the calculations of DEA efficiency. The result of our efforts is an *LUs efficiency map* that can help regional planners improve the potential supply of ESs in inefficient LUs by looking at the LULC composition of the most efficient LUs, corresponding ESs, and the associated costs.

For the sake of argument and to verify the spatial distribution of ESs on different groups of LULCs, the results were summarized and contrasted within *landscape types*, as suggested by Hladnik (2005). The typology chosen presents the results from a different perspective that takes into account terrain features (e.g., forests are primarily located on higher and steeper terrains, which are less suitable for agriculture; Hladnik 2005). He distinguished three landscape types: (a) *forest landscapes* are landscapes with a forest percentage of more than 85%, (b) *forested landscapes* are landscapes with a forest percentage between 40 and 85%, and (c) *agricultural and urban landscapes* are landscapes with a forest percentage of less than 40%. To test whether there are statistically significant differences between two or more groups (e.g., ES groups, LULCs, landscape types), we used the *Kruskal-Wallis H test*; to show the correlation between ESs, landscape types, and LULCs, we used *Spearman’s rank correlation coefficient*.

### Analyzing drivers of change through regression analysis

Finally, Tobit regression was used to assess the underlying drivers of change that affect the efficiency of LUs. Tobit regression is a special case of censored regression, in which the dependent variables are bounded from below, above, or both (Hoff 2007). We opted for a two-limit Tobit regression analysis since the efficiency scores were bounded between zero and one. As noted by Ramalho et al. (2010), Tobit and linear regression (ordinary least squares) models are traditionally used to regress efficiency analysis results. The Tobit model assumes the existence of a latent variable, y^*^_i_, which is unobservable and is hypothesized to underlie the observed dependent variable scores. As pointed out by Hoff (2007), the Tobit model is a misspecification for second-stage efficiency analysis with DEA, yet it is sufficient for modeling DEA efficiency values against independent variables. The DEA efficiency value is defined by several environmental variables (*x*_*i*_) and a latent random variable (y^*^_i_), as shown in Eq. 5.

5 $$\begin{array}{cc}{y}_{i}^{* }={x}_{i}\beta +{\varepsilon }_{i} & {\rm{with}}\,{\varepsilon }_{i}\,\approx\, N\left(0,{\sigma }^{2}\right)\end{array}$$

where *x*_*i*_ is a 1 × *k* vector of observations on the constant and *k* – 1 efficiency factor explanatory variables, *β* is a k × 1 vector of unknown coefficients, and *ε*_*i*_ is the random error term that is normally distributed with mean 0 and variance *σ*^*2*^ (McDonald 2009). If y^*^_i_ ≤ 0, the efficiency score for the *i*th production unit, *y*_*i*_ = 0, if y^*^_i_ ≥ 1, *y*_*i*_ = 1, and if 0 < y^*^_i_ < 1, *y*_*i*_ = y^*^_i_

Using this latent variable y^*^_i_, the efficiency values *y*_*i*_ are defined as censored above 1 and below 0. This means that if y^*^_i_ is greater than 0, we observe the actual value of *y*_*i*_, and if y^*^_i_ is less than or equal to 0, we observe a censored value of 0. Likewise, if y^*^_i_ is greater or equal to 1, we observe the censored value of 1, otherwise, the actual value of *y*_*i*_. Tobit regression models censored dependent variables by explicitly accounting for an unobservable latent variable that influences the observed outcomes, thereby providing insights into the factors contributing to the observed distribution. Tobit regression analysis was performed using *Gretl 2022a* software (^©^ Allin Cottrell and Riccardo “Jack” Lucchetti).

Based on the literature reviewed and survey results, we chose to analyze the impact of seven drivers of change that may influence the efficiency of LUs. The main criterion for the selection of driving factors was that the factor has already been recognized and described by the research community. The second important criterion for including variables was the ability to obtain spatially explicit data with some variation. The second criterion is a severe restriction that disables the inclusion of several interesting variables such as average income, education, life satisfaction, and community-level economic development indicators. We followed the classification of factors affecting landscape change proposed by Bürgi et al. (2004) and divided them into five groups: *socioeconomic* (population density, number of different LULCs), *natural* (average slope, average elevation), *political* (proportion of Natura 2000 area), *technological* (road density), and *cultural* (distance to cultural heritage sites). We hypothesized that lower population density would increase the efficiency of LUs by positively affecting the heterogeneity of LULC use and expressing synergies among ESs (Feng et al. 2021). Furthermore, we hypothesized that a higher proportion of Natura 2000 network, higher road density, higher number of different LULCs per square kilometer, greater distance from cultural heritage sites, greater slopes, and higher average elevation would also increase LUs efficiency (Schirpke et al. 2013; Paracchini et al. 2014; Allen 2015; Rolo et al. 2021; Sasaki et al. 2021; González-García et al. 2022). The spatially explicit slope and elevation data were extracted from a digital elevation model with a resolution of 10 m. The number of different LULCs was calculated from the spatially explicit LULC map provided by the Ministry of Agriculture, Forestry, and Food. Other maps were retrieved from national authorities: the Ministry of Environment and Spatial Planning for Natura 2000 maps, the Survey and Mapping Authority for road network maps, and the Ministry of Culture for cultural heritage sites. All data refer to the state in 2020.

## Results

### Potential supply of ESs

**Fig. 3:**
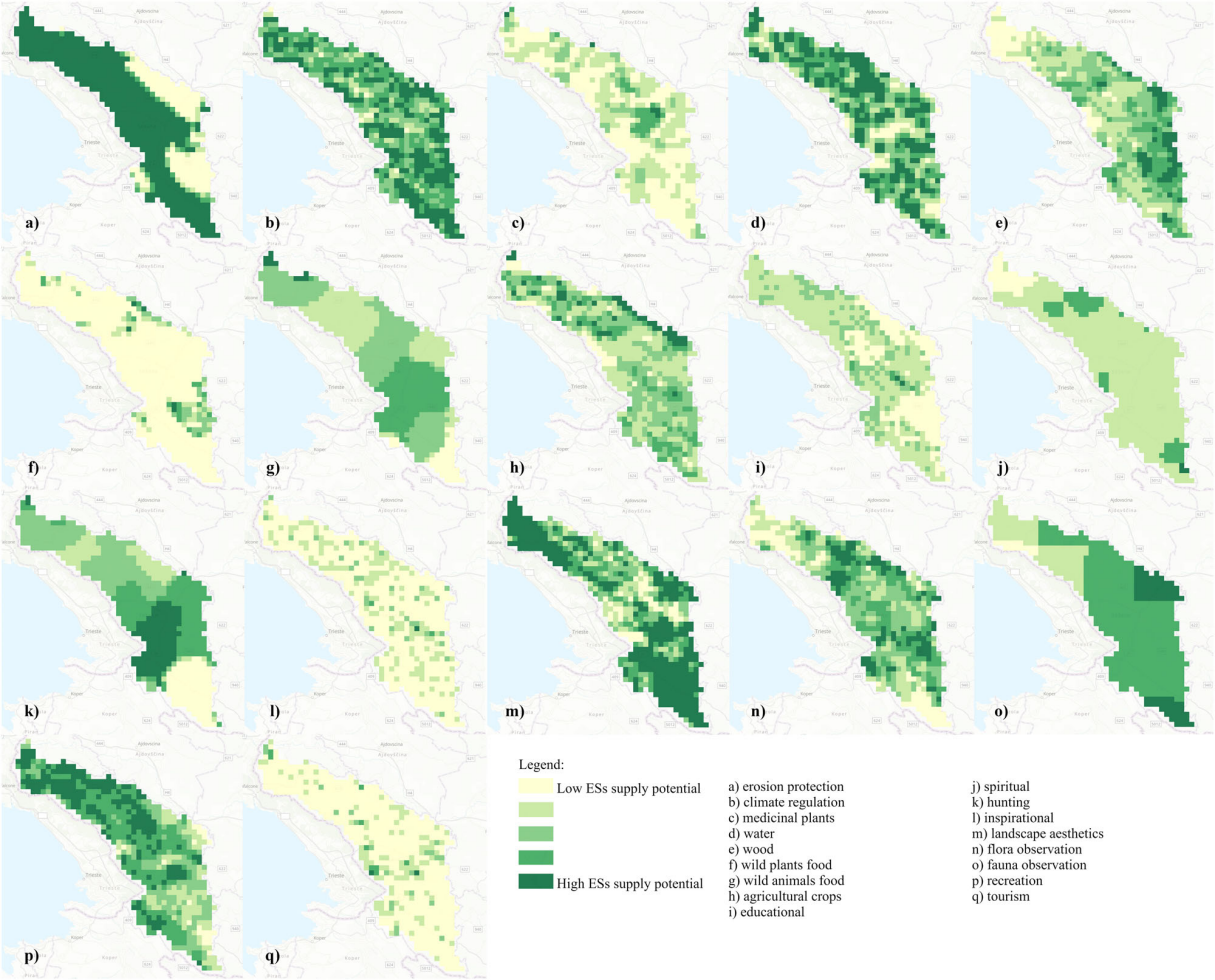
Maps of the potential supply of ES: **a** erosion protection, **b** climate regulation, **c** medicinal plants, **d** water, **e** wood, **f** wild plants food, **g** wild animals food, **h** agricultural crops, **i** educational, **j** spiritual, **k** hunting, **l** inspirational, **m** landscape aesthetics, **n** flora observation, **o** fauna observation, **p** recreation, **q** tourism (own elaboration; map sources: ESRI, authors)

The spatially explicit ESs assessment yielded 17 maps with the potential supply of ES, shown in Fig. 3. While some maps are rather homogeneous at a larger scale (e.g., erosion, fauna observation, and wild animal food), most maps reflect the potential supply of ES in a heterogeneous and distributed manner at a smaller scale (e.g., wild plant food, tourism, and inspirational).

To investigate whether there are differences between ESs in different landscape types, as proposed by Hladnik (2005), the Kruskal-Wallis H statistic was used. The results are presented in Table 2. Except for ES climate regulation, which is significantly strongly positively associated with forest landscape type, no very strong positive relationships between landscape types and ESs can be observed. This feature is due to the chosen modeling approach, which results in weak or medium relationships between the different LULCs. However, there are significant differences in the combination of ESs groups among the different landscape types, as shown by the Kruskal–Wallis *H* statistic (last column of Table 2). The vast majority of cultural ESs relate to landscapes with a lower proportion of forests, and there are statistically significant differences with other landscape types. ES fauna observation is an exception and a meaningful one since the wildlife included in the analysis live primarily in forests.

**Table 2:** Spearman’s rho correlations between individual ESs and landscape types.

Ecosystem service	ES group	Forest landscape (≥85% forest)	Forested landscape (≥40% to <85% forest)	Agricultural and urban landscape (<40% forest)	Kruskal–Wallis *H*-test
Erosion protection	Regulating	−0.121**	−0.002	0.130**	15.469 ***
Climate regulation		0.712***	0.100*	−0.625***	439.672 ***
Hunting	Cultural	0.019	−0.052	**0.044**	1.908 (p = 0.385)
Flora observation		−0.097*	0.036	**0.058**	6.590 *
Tourism		−0.314***	0.055	0.262***	82.070 ***
Recreation		−0.277***	0.171***	0.081*	48.034 ***
Inspirational		−0.330***	0.063	0.268***	88.853 ***
Education		−0.180***	0.037	0.144***	26.251 ***
Landscape esthetics		0.083*	−0.095*	0.030	6.177 *
Fauna observation		0.156***	−0.131**	−0.003	16.429 ***
Spiritual		0.007	−0.026	**0.024**	0.504 (p = 0.777)
Wood	Provisioning	0.139***	−0.070	−0.060	12.325 **
Wild plants food		0.079*	0.051	−0.146***	14.342 ***
Wild animal food		0.036	0.062	−0.114**	8.168 *
Crops		−0.086*	−0.079*	0.187***	22.638 ***
Medicinal		−0.120**	**0.060**	0.053	9.247 *
Water		−0.171***	0.019	0.157***	26.441 ***

*p < 0.05, **p < 0.01, ***p < 0.001

Bold font indicates the ES’s highest positive correlation

Similar characteristics are expressed by ES landscape esthetics and hunting; both are positively related to either much forest or much agricultural and other non-forest areas. However, all the above correlations are rather very weak. On the other hand, there is a significant relationship between forests and ES wood production, and ES climate regulation. Both relationships are meaningful because wood production occurs in forests, and forest soils store more carbon than other LULCs (e.g., Schirpke et al. 2013; Table A.1). Similarly, ES crop production is positively related to areas that have a higher proportion of agricultural land, as crop production mainly extends to agricultural LULCs.

ES wild plant’s food gathering also relates with forest and forested landscapes but little with agricultural land. Chestnut growing stock - that is the proxy variable for this ES - occurs in forests, but since it is rather sparsely distributed in the study area (in other words, it does not form large forest stands), the relationship with forests is rather weak and not significant. On the other hand, ES medicinal plants increase with increasing settlement size, and larger settlements have a considerable proportion of non-forest LULCs. However, meadows, sparsely vegetated lands, and transitional lands (e.g., old field succession) remain the main source of medicinal plants.

In addition, we also wanted to examine whether the three ES groups (i.e., provisioning, cultural, and regulating) showed statistical differences across the three landscape types. To this end, we calculated the average values of the normalized ESs that fall into one of the three groups (see Table 2 for the groups and the ESs they contain). The average values are shown in Table 3, with the standard deviation in parentheses and with the Kruskal-Wallis H statistic in the last column. The higher the average value, the higher the potential supply of ESs. Cultural ESs values increase with decreasing forest cover, and this difference is statistically significant (*H*(2) = 19.330, *p* < 0.001). This indicates that, on average, these ESs are more likely to be found near settlements, which are often located near agricultural land. Similarly, the potential supply of all ESs increases on average with decreasing forest cover (*H*(2) = 9.468, *p* = 0.009). Regulating ESs values are highest in forested areas, followed by forest, agricultural, and urban areas (*H*(2) = 60.239, *p* < 0.001). Finally, the values of provisioning ESs are best in agricultural, urban, and artificial areas, followed by forests and forested areas (*H*(2) = 4.046, *p* = 0.132). However, the differences are not statistically significant, leading to the conclusion that all areas, regardless of the proportion of different LULCs, supply the potential amount of provisioning ES equally well.

**Table 3:** Averaged ESs values between different landscape types and Kruskal–Wallis’ statistic (number in parentheses is standard deviation)

ES group/landscape type	Forest landscape (≥85% forest)	Forested landscape (≥40% to <85% forest)	Agricultural and urban landscape (<40% forest)	Kruskal–Wallis *H*-test
Regulating ES	79.407 (23.855)	80.474 (20.156)	76.618 (17.523)	60.239 (*p* < 0.001)
Cultural ES	38.223 (4.593)	39.186 (5.705)	41.003 (5.097)	19.330 (*p* < 0.001)
Provisioning ES	32.934 (7.949)	32.881 (6.399)	33.862 (6.856)	4.046 (*p* = 0.132)

### Efficiency of LUs

**Fig. 4:**
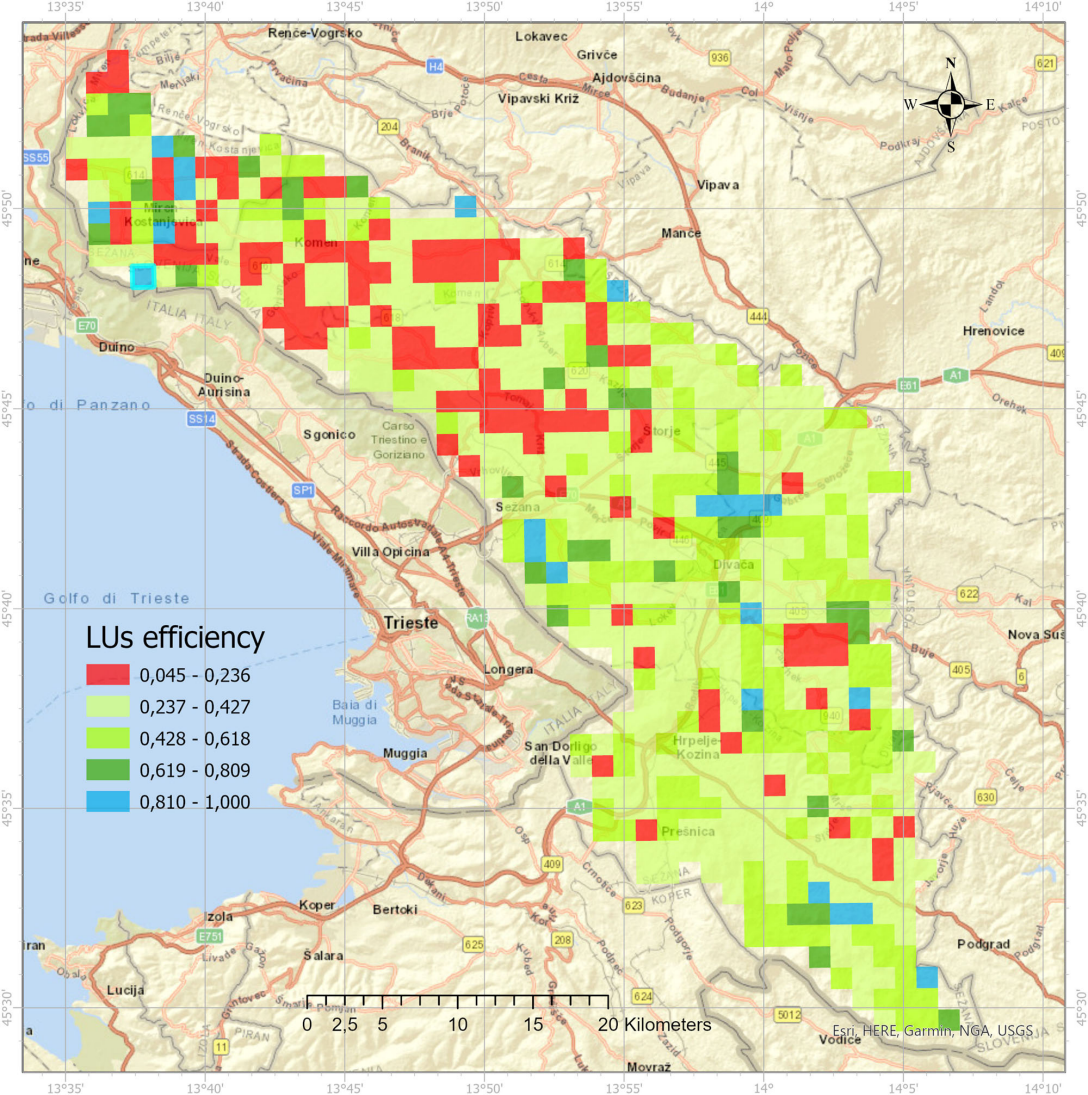
LUs efficiencies mapped (own elaboration; map sources: ESRI, authors)

The following section alternately reports the potential supply of ESs, mapping, and analysis results at the level of the LUs, which consider the average management costs. In other words, the raw ESs values were “weighted” by the management costs for each LULC within a given LUs. The average efficiency of the LUs was 0.391 (st. dev. = 0.178), and 12 LUs were efficient (i.e., they received an efficiency score of 1). Of the 615 inefficient LUs, 41.6% of LUs scored above average efficiency (i.e., they are *less inefficient*), and 58.4% of LUs scored below average efficiency (i.e., they are *more inefficient*). The LUs with the lowest efficiencies (0.045 and 0.071) were in the urbanized and agriculturally intensive areas in the far northwest (Fig. 4). Other inefficient LUs also include areas with a low proportion of forests and a high proportion of agricultural and urban landscape types. In fact, the forest landscape type is characteristic of higher efficiency LUs, and the agricultural and urban landscape type is found in the most inefficient LUs.

Efficient LUs include some of the famous places (e.g., Škocjan Caves Regional Park, Lipica, Cirje Monument) in the study area. In addition, LUs such as those south of the town of Sežana are efficient, although they have relatively low values for several ESs. Eleven out of seventeen ESs exhibit higher average values on efficient LUs, and the average management costs are three times lower than the average management costs of inefficient LUs, so these LUs are efficient. Average ESs values that are higher in inefficient LUs are timber production, water, spiritual, flora observation, and fauna observation. In turn, other LUs are in areas with 100% forest cover or in close proximity to these areas. On average, the 12 efficient LUs include the least amount of urban and artificial land than inefficient LUs (Table 4). Similarly, water bodies are characteristic of efficient LUs and inefficient LUs, as opposed to less inefficient LUs. More inefficient LUs have a significantly higher average proportion of pastureland (*H*(2) = 91.114, *p* < 0.001), agricultural fields (*H*(2) = 142.647, *p* < 0.001), permanent crops (*H*(2) = 114.143, *p* < 0.001), and old field succession (*H*(2) = 22.919, *p* < 0.001) than efficient and less inefficient LUs. The proportion of other non-agricultural areas is not significantly different among the three types of efficient LUs (*H*(2) = 5.475, *p* = 0.065). The noteworthy observation that more inefficient LUs exhibit a higher average proportion of pastureland, agricultural fields, permanent crops, and old field succession prompts a valuable exploration into the implications of these LULCs on the supply of ESs. Pastureland and agricultural fields, characterized by intensive human activities, may contribute to increased land management challenges, potentially leading to a diminished supply of ES. Permanent crops, although providing certain benefits, might incur higher maintenance costs, affecting overall efficiency. Additionally, old field succession areas could indicate areas with less active land management, influencing the quality and quantity of several ESs (e.g., carbon sequestration). Factors contributing to this LULC composition and consequent LUs inefficiency may include poor land use planning, suboptimal agricultural and forestry practices, or insufficient conservation efforts.

**Table 4:** Average shares of LULCs between different LUs efficiency groups and Kruskal–Wallis’s statistic

	Efficient (%)	Less inefficient (%)	More inefficient (%)	Kruskal–Wallis *H*-test
Forests	78.84	73.42	54.98	94.957 (*p* < 0.001)
Pastureland	8.95	16.15	27.51	91.114 (*p* < 0.001)
Old-field succession	6.72	2.96	2.96	22.919 (*p* < 0.001)
Other agricultural areas	2.77	3.41	5.33	73.239 (*p* < 0.001)
Urban and artificial areas	1.4	3.23	4.81	52.036 (*p* < 0.001)
Other non-agricultural areas	0.6	0.2	0.11	5.475 (*p* = 0.065)
Permanent cultures	0.41	0.42	2.67	114.143 (*p* < 0.001)
Fields and gardens	0.18	0.19	1.52	142.647 (*p* < 0.001)
Water bodies	0.14	0.02	0.1	14.395 (*p* < 0.001)

According to Table 4, the most efficient distribution of LULCs for an average LU is defined by a relatively high proportion of forests (78.84%), followed by relatively low pastureland (8.95%) and high old-field succession (6.72%). The remaining 5.49% of LULCs include other agricultural areas (2.77%), urban and artificial areas (1.40%), other non-agricultural areas (0.60%), permanent crops (0.41%), fields and gardens (0.18%), and water bodies (0.14%). In this setting, LUs supply the most ESs with the lowest management costs but not necessarily all or the most absolute ESs. This is clear from Table 5, where regulating and cultural ESs are highest in efficient LUs, but provisioning ESs are highest in less inefficient LUs. The significantly different mean values of regulating (*H*(2) = 10.725, *p* = 0.005) and cultural (*H*(2) = 9.168, *p* = 0.010) ESs between efficiency categories indicate that efficient LUs are indeed highly dependent on ESs from these ES groups. On the other hand, the differences in provisioning ESs (*H*(2) = 3.659, *p* = 0.160) and overall ESs (*H*(2) = 4.022, *p* = 0.134) are not statistically significant, so these ESs remain more or less the same between the LUs efficiency categories.

**Table 5:** Averaged ESs groups values between three LUs efficiency groups and Kruskal-Wallis’s statistics (number in brackets is the standard deviation)

ES group/LUs efficiency group	Efficient (*n* = 12)	Less inefficient (*n* = 256)	More inefficient (*n* = 359)	Kruskal–Wallis *H*-test
Regulating ES	92.109 (14.722)	77.712 (21.949)	80.229 (19.633)	10.725 (*p* = 0.005)
Cultural ES	59.690 (21.443)	58.113 (7.833)	59.598 (7.514)	9.168 (*p* = 0.010)
Provisioning ES	19.972 (7.576)	22.370 (4.905)	21.915 (4.210)	3.659 (*p* = 0.160)
All ES	42.572 (8.651)	41.496 (4.115)	42.075 (3.811)	4.022 (*p* = 0.134)

### Exploring drivers of change that affect LUs efficiency

Before running the regression model, we checked Spearman’s rho correlations between the dependent and independent variables to estimate their relationships and check for possible multicollinearities between the variables. Only variable *Number of different LULCs* is strongly and statistically significantly correlated with efficiency outcomes (*r*_s_ = −0.519, *p* < 0.001). Similar, but weaker, is the relationship with the variables *Road network density* (*r*_s_ = −0.326, *p* < 0.001) and *Share of Natura 2000 sites* (*r*_s_ = −0.105, *p* = 0.008). The relationship between efficiency results and the variables *Average slope* (*r*_s_ = 0.242, *p* < 0.001) and *Distance to cultural heritage sites* (*r*_s_ = 0.280, *p* < 0.001) is positive, statistically significant, and weak. The relationship between efficiency scores and the variable *Average elevation* is also positive, statistically significant, and moderate (*r*_s_ = 0.305, *p* < 0.001). Despite some independent variables exhibiting strong correlation, the maximum Variance Inflation Factor of independent values of the build model did not exceed the value of 1.870. Therefore, we concluded that no multicollinearity issues whatsoever are found in the model.

A Tobit model was built using the *Enter method* and included all covariates. The results are presented in Table 6. There were four statistically significant covariates in the model, namely *population density*, *road density*, *average slope*, and *number of different LULCs*. Out of the selected and analyzed covariates, these four variables are the most important drivers of LUs efficiency. In particular, the number of different LULCs and road density have the highest coefficients. The results show that for an additional different LULC in a LU and a denser road network, the average efficiency decreases by 2.63% and 0.11%, respectively. On the other hand, efficiency increases by 0.10% and 0.16% when adding one person per square kilometer and increasing the gradient by one percent, respectively. Particularly, population density was unexpectedly positively affecting efficiency. A possible explanation of this finding lies in the relatively low average population densities and traditionally sparse distribution of built areas in the study region. The other three covariates also have a positive effect on the efficiency results but are not statistically significant.

**Table 6:** Tobit regression analysis results

Covariate	Covariate group	Coefficient	Std. error	*z*	*p*-value
Constant	/	59.2923	5.9341	9.992	<0.001***
Population density	Socio-economic	0.0950	0.0167	5.696	<0.001***
Share of Natura 2000 sites	Political	0.0197	0.0210	0.936	0.349
Road density	Technological	−0.1064	0.0268	−3.966	<0.001***
Average slope	Natural	0.1702	0.0727	2.341	0.019**
Average elevation	Natural	0.0001	0.0057	0.011	0.991
Number of different LULCs	Socio-economic	−2.6334	0.3553	−7.411	<0.001***
Distance to heritage sites	Cultural	0.0014	0.0023	0.625	0.532

Although the Tobit analysis yields a pseudo-*R*^2^ value rather than a “real” *R*^2^ determination coefficient, the squared correlation between fitted and original values can provide a rough measure of the quality of the model (Kapfer et al. 2012). With the adjustment described above, the model was able to explain 27.9% of the variation in efficiency by variation in the seven independent variables. In general, more densely populated LUs with lower road densities have higher efficiency than the opposite. Similarly, LUs located on steeper slopes with homogeneous landscapes are more efficient with respect to different LULCs than flatter and more heterogeneous LUs. The statistically non-significant coefficients of the variables are all positive, implying that efficiency scores increase with increasing proportion of Natura 2000 sites, increasing average elevation, and increasing distance from cultural heritage sites, although this increase is not significant in individual LUs.

## Discussion

The study area is a traditional cultural landscape with heterogeneous ecosystems, diverse ESs, and high species diversity. The Karst region has been inhabited for a long time, with evidence of human presence dating back to prehistoric times (Zorn et al. 2015). Over the centuries, various cultures and civilizations have left their mark on the region, reflecting in the current heterogeneous ecosystems, high species diversity, and a high potential to supply various ESs. In addition to being ecologically rich, the region has valuable historical and cultural value, making it an interesting and suitable study area for the study of ESs and their relations with landscapes in terms of efficiency.

In our study, seventeen ESs were mapped, using data ranging from very local (e.g., tourism) to broad and widespread (e.g., faunal observations) at a resolution that resulted in heterogeneous or homogeneous patterns, respectively. Several ESs can be associated with specific LUs. The observed positive correlation between cultural ESs and agricultural landscapes (i.e., areas with less than 20% forest cover) underscores the connection between cultural values and open, agriculturally dominated environments. This association is indicative of the enhanced accessibility of cultural ESs in regions with better infrastructure and closer proximity to settlements. Agricultural landscapes, featuring human-centric elements and historical significance, become easily accessible spaces for recreational and cultural activities. The positive relationship highlights the appeal of these areas for cultural ESs, as individuals can readily engage with it and appreciate the aesthetic and recreational aspects of the landscape. This alignment with the notion that cultural ESs are more frequently utilized in regions with improved infrastructure or near settlements, as suggested by Queiroz et al. (2015), emphasizes the practical considerations, such as accessibility, that significantly contribute to the utilization of cultural ESs in certain LUs.

Erosion protection is also related to agricultural and urban landscapes, which can be explained by the higher-than-average slopes and hilly terrain of forest-dominated areas. The relatively low correlations between most ESs and landscape types suggest that the mapped ESs do not have much to do with a particular ecosystem but are generally distributed across the landscape. As pointed out by Raudsepp-Hearne et al. (2010), this could be further supported by the notion of complex patterns of ESs generated in a social-ecological system. On the other hand, ES climate regulation is greater in areas with higher forest cover, as forest soils store more carbon than other ecosystems (e.g., Fernandez-Campo et al. 2017). Regarding provisioning ES, these are also unevenly distributed across landscape types, but for obvious reasons, the provision of wood products and agricultural crops are strongly associated with forests and agricultural land, respectively.

Compared to the ESs values for the landscape types, the efficiency values for the LUs show different trends. This is due to the inclusion of management costs (i.e., inputs) that “balance” the relationship between inputs and outputs (i.e., costs and benefits) within the LUs. This leads to the conclusion that when costs are included, LUs with certain ESs are represented in an “economically acceptable” manner. In other words, the LUs efficiency map created acts as a map of ESs acceptability or a map of hot and cold spots to show, for example, LUs that must increase their levels of ESs to become efficient. Efficient LUs have a LULC composition that delivers the most ESs at the lowest management cost. This composition includes relatively more forests, successional lands, other non-agricultural areas, and water bodies than other inefficient LUs. While this is true for our study, we are aware that other specificities might influence the LULC composition of LUs, such as the degree of human intervention (e.g., Kovács et al 2020). On the other hand, fields and gardens, pastureland, and permanent cultures (all are rather cost-intensive LULCs) exhibit significantly lower shares. Given the relatively low average cost of forest management and the high cost of agricultural production, it is therefore important to note that forests and forested landscapes are better in terms of the efficiency of LUs. Forests tend to be long-lived ecosystems and require the fewest inputs, giving them an “efficiency advantage” (conf. competitive advantage). Yet, at the same time, these LUs with more forested areas may not supply other ESs, e.g., provisioning ES (see also Table 5) to such an extent. This result is consistent with previous findings that have established the relative importance of heterogeneous landscapes for the provision of different ESs (e.g., Rolo et al. 2021).

While the arguments for the high proportion of forests and other non-agricultural areas have been presented above, the high proportion of old field succession can be explained by the relatively high importance of ESs and biodiversity, as suggested by (Sasaki et al. 2021). These areas tend to be located on marginal land and away from settlements, as they are more likely to be located on unfavorable terrain conditions. From an efficiency perspective, old-field succession is set-aside land, and therefore does not incur direct management costs, but potentially supplies high ESs. Similarly, water bodies in a landscape are generally beneficial for several ESs, from water purification in wetlands (Turner et al. 2014) to water quality (Queiroz et al. 2015) to recreation (Paracchini et al. 2014).

The majority of inefficient LUs are found near and around smaller villages. In contrast, LUs located near larger towns (e.g., Sežana) have higher than average efficiency, indicating the relatively “expensive” configuration of the traditional landscape structure with settlements surrounded by agricultural fields. This does not mean that these villages are not livable, but from the perspective of ESs supply and associated costs, they tend to be inefficient. In other words, the potential benefits of denser urban and human-made areas surrounded by forested land are higher per unit cost than thinner settlements surrounded by agricultural land. Importantly, while most ESs are potentially consumed by society as a whole—many of which are essentially free to them (e.g., mushroom picking)—the costs to society are very small. In fact, most of the direct costs of ecosystem conservation remain on the shoulders of landowners or land managers. This goes hand in hand with the findings of Vejre et al. (2015), which showed that ESs related to cultural landscapes are often required at the landscape level, regardless of ownership, but are supplied by a system of individual private lands. Nonetheless, the government plays a significant role in helping landowners redistribute these additional costs to society through incentives (e.g., subsidies) and other policies.

In the final phase of our study, we conducted a comprehensive regression analysis to assess the potential impact of seven drivers of change on LUs efficiency. Our findings reveal that LU efficiency decreases with increasing road density and landscape heterogeneity, as measured by the number of distinct LULCs per km^2^. The moderate correlation between these two variables suggests that remote areas exhibiting greater landscape homogeneity are less accessible due to lower road density. This accessibility limitation implies that such LUs, also characterized by a higher proportion of forests and consequently lower management costs compared to agricultural regions, might face challenges in supplying some ESs. Conversely, lower population density and a gentler average slope are associated with lower LUs efficiency, indicating that LUs are more efficient in providing ESs in areas with human presence or higher topographic diversity. This contrasts with the findings of Feng et al. (2021), who, employing Bayesian inference, asserted that certain land use types (e.g., forests), higher slopes, higher vegetation coverage, and lower population density positively influence ESs and their interactions. The disparities between our results and those of Feng et al. (2021) underscore the nuanced interplay of factors influencing LUs efficiency, emphasizing the need for context-specific interpretations and the consideration of diverse ecological and geographical settings in land management planning. Three variables in the model were found not to be statistically significant, namely the share of Natura 2000 sites, distance to cultural heritage sites, and average elevation. Nevertheless, their coefficients are positive, suggesting that the presence of Natura 2000, more distant cultural heritage sites, and higher elevation increase the efficiency of LUs. In terms of the model’s explained variability in efficiency (i.e., 27.9%), our model shows significantly lower values compared to other studies that used the same two-stage approach, combining DEA results with regression analysis. Since the variability largely depends on the selection and quality of the independent variables, this lower explained variability can be further improved by adding other factors or improving their quality.

The results of the study could be extremely useful for regional planners, policymakers, local residents, and the public. Identification of inefficient areas provides a roadmap for targeted conservation and restoration efforts, allowing regional planners to prioritize areas for conservation and focus resources on inefficient locations. Understanding the role of landscapes in providing ESs can be used for community engagement and education, which can foster a sense of stewardship and compliance with conservation efforts. Policymakers can rethink and align the regulations and mechanisms to encourage sustainable practices in those areas and design outreach programs to educate communities about the value of local ecosystems. This can further foster a sense of stewardship and compliance with conservation efforts.

The conceptual and methodological framework presented could be useful for similar regions and situations around the world. In particular, the methodology described does not require extensive data collection, as many of the spatially explicit data layers can be found on the World Wide Web or accessed or modeled using freely available software. Participatory approaches should also be used when no data are available at all or when one wishes to increase the legitimacy of the results. In either case, failure to consider traditional ecological knowledge and the real needs of local residents leads to inefficient LUs and unmet human needs. Framework’s downside is its static and deterministic condition analysis which yielded limited results. Since the delivery of some ESs changes between seasons and generally in time, our study cannot offer an otherwise important aspect.

### Caveats and limitations

In reality, we do not know what the cost of supplying ESs is. Therefore, in this study we have assumed that the cost of potentially supplying ES is proportional to the cost of land use management. This may not be the case, as the supply of some ES (e.g., tourism) may require additional costs to establish infrastructure. The simplification of input parameters may have an impact on the accuracy and bias of some ES values over others, potentially resulting in underestimation of costs by not considering specific management costs for particular ESs. To address these issues and reduce uncertainties in the results, it may be beneficial to invest more effort into developing improved proxies and suitable methodologies to measure them. These methods include participatory GIS, social media data analysis, probabilistic methods, spatial statistics, data mining, machine learning approaches, and others.

An important consideration for a spatially explicit analysis is the availability and reliability of spatially explicit data. Since this is a necessary condition for conducting such analyses, in the absence of appropriate data for specific inputs, extensive data collection should be conducted to build even more accurate models. Spatial data quality can vary significantly, affecting the accuracy of analyses and models. However, using freely available data is cost-effective and time-efficient, despite the data may lack rigor and legitimacy of data collected through well-designed studies. In our study, therefore, we wanted to explore the possibilities of readily available and freely usable data from various agencies to perform such an analysis at the landscape level. In this context, we are aware of the problems that this strong constraint poses, particularly in terms of the accuracy and legitimacy of the results. On the other hand, relaxing this constraint would require either a reduction in valuable spatial resolution or the implementation of expensive and time-consuming explicit spatial data collection. In conclusion, the challenges related to spatial-explicit data underscore the importance of accurate and legitimate spatial data. The trade-offs between readily available data and extensive data collection involve considerations of cost, precision, timeliness, and data legitimacy. These factors depend on the specific requirements and objectives of the modeling or analysis efforts.

Similarly, we are aware of the limitations the deterministic models have; including stochasticity or selecting other variables would yield different results. As for DEA, it represents an intuitive method that is free of units and computes a single-value result that is easy to interpret. One of the great advantages is the flexibility of the concept of DMU, which originally represented business units of analysis such as companies, firms, and organizations (Kapfer et al. 2012; Susaeta et al. 2016), but can take very many forms, such as administrative units, natural boundaries, ecosystems, pedological units, species, and others. On the other hand, it is important to keep in mind that it is also a deterministic method that is sensitive to the selection of inputs and outputs. Potential research directions and alternatives to DEA encompass the use of stochastic methods (e.g., Stochastic Frontier Analysis), Bayesian inference (e.g., Bayesian DEA), or adding machine learning algorithms (e.g., Artificial Neural Networks). Moreover, it is not spatially explicit in terms of accounting for surrounding units in the calculations. In order to take into account the spatial distribution, spatial autocorrelation (e.g., local Moran’s I) should have been joined with DEA analysis. Since all ESs may not be available in all seasons and decades, future studies should consider the temporal dimension (e.g., via Malmquist indices) in combination with more sophisticated data-driven methods (e.g., machine learning). Ideally, including the temporal dimension would allow one to study the reliability of ESs supply as well as the capacity of ecosystems to adapt to various drivers of change. Other types of regressions to examine the influence of drivers of change might also be worth considering (see e.g., McDonald 2009). Importantly, we are aware of the limited selection of drivers of change. Expanding the list of spatial explicit drivers of change would potentially yield different and interesting results worth exploring. Finally, adding information on the demand side of ESs would further strengthen the results and contribute positively to sustainable resource management.

## Conclusion

In this study, we used a three-step approach to identify the efficiency of LUs for providing landscape-level ESs. After creating seventeen maps of the potential supply of ESs, we calculated the relative efficiency of LUs to identify hot and cold spots for the potential supply of ESs. To evaluate the relative efficiency of LUs and given the problems of size imbalance, multiple inputs, and associated computational limitations, we used the DEA method. LUs are basically represented as a composition of different ecosystems that incur certain management costs and provide various ESs and benefits to people. To examine the effects of drivers of change on LUs efficiency, we regressed LUs efficiency on seven recognized drivers of change using Tobit regression. Following the conceptual framework developed and using this method to account for the cost of supplying ESs, we identified the most efficient LUs with a given LULC composition and determined how much the drivers of change contribute to that efficiency. The study concludes with two important findings: First, forests play a critical role in supplying many ESs and remain the cornerstone for the efficient supply of ES on any landscape, particularly because of their relatively low management costs and large spatial extent. Second, justifying the potential supply of multiple ESs by considering the various (management) costs increases the reality of the result’s likelihood of public acceptance and legitimizes the choices made. Future research can delve deeper into understanding the dynamics and interactions of ESs, exploring how changes in landscape management practices impact the supply of ESs and investigating the trade-offs and synergies to develop integrated management strategies that optimize multiple ESs simultaneously. Finally, taking into account the effects of climate variations on ESs would facilitate more climate-resilient landscape planning.

### Supplementary information

Supplementary_revised
