# Supplementary material for: Investigating potential supply of ecosystem services in cultural landscapes through efficiency analysis
Source: Environ Manage. 2024 Apr 11;74(2):161–79. doi: 10.1007/s00267-024-01967-5 (PMC11227448; doi:10.1007/s00267-024-01967-5)
Supplement: Supplementary file 1 — Supplementary_revised [file 267_2024_1967_MOESM1_ESM.docx]

# Appendices

Appendix A.1

Table A.1: Carbon stock in soils for different LULC

| Land Use/Land Cover | LULC code | Carbon stock in soils [Mg C ha^-1^] | Source |
| --- | --- | --- | --- |
| Field | 1100 | 89,8 | (Šinkovec et al. 2021) |
| Permanent crop | 1180 | 89,8 |  |
| Greenhouse | 1190 | 89,8 |  |
| Vineyard | 1211 | 63,7 |  |
| Intensive orchard | 1221 | 71,5 |  |
| Extensive orchard | 1222 | 90,5 |  |
| Olive tree plantation | 1230 | 90,5 |  |
| Other permanent plantation | 1240 | 71,5 |  |
| Pasture | 1300 | 92,8 |  |
| Agricultural land with trees | 1800 | 99,1 |  |
|  |  |  |  |
| Old-field succession | 1410 | 99,1 |  |
| Forest plantation | 1420 | 99,1 |  |
| Trees and shrubs | 1500 | 118,1 |  |
| Uncultivated land | 1600 | 89,8 |  |
|  |  |  |  |
| Urban and artificial area | 3000 | 0 | (Raudsepp-Hearne et al. 2010) |
| Dry open areas with special vegetation | 5000 | 0 |  |
| Open areas with no or minimum vegetation | 6000 | 0 |  |
| Water body | 7000 | 95,2 | (Buosi et al. 2021) |
| Forest | 2000 | 120,9 | (Skudnik et al. 2021) |

Appendix A.2

Table A.2: LULC types, groups and corresponding management costs

| LULC | LULC code | LULC group | Management costs (€ ha^-1^) | Description |
| --- | --- | --- | --- | --- |
| Field | 1100 | Fields and gardens | 13,616.90 € | average costs of producing various crops |
| Permanent crop | 1180 |  | 6,657.55 € | average costs of producing various crops and plantations |
| Greenhouse | 1190 |  | 52,949.25 € | average costs of producing crops in greenhouses |
| Vineyard | 1211 | Permanent cultures | 7,371.00 € | costs of vineyards |
| Intensive orchard | 1221 |  | 16,132.00 € | average costs of apple, peaches and pear orchards |
| Extensive orchard | 1222 |  | 13,828.00 € | average costs of producing apples, peaches, pears and olive trees |
| Olive tree plantations | 1230 |  | 6,636.91 € | costs of olive trees |
| Other permanent plantation | 1240 |  | 11,516.76 € | average costs of apples and pears orchards, vineyards and olive trees |
| Pasture | 1300 | Pastureland | 1,026.02 € | average costs of managing pastures for hay and silage |
| Agricultural land with trees | 1800 |  | 950.99 € | average costs of forest management and pasture |
| Old-field succession | 1410 | Old-field succession | 0.00 € | / |
| Forest plantation | 1420 | Other agricultural land | 725.91 € | costs of forest management |
| Trees and shrubs | 1500 |  | 0.00 € | / |
| Uncultivated land | 1600 |  | 0.00 € | / |
| Forest | 2000 | Forests | 725.91 € | costs of forest management |
| Urban and artificial area | 3000 | Urban and artificial areas | 0.00 € | / |
| Dry open areas with special vegetation | 5000 | Other non-agricultural land | 0.00 € | / |
| Open areas with no or minimum vegetation | 6000 |  | 0.00 € | / |
| Water body | 7000 | Water bodies | 0.00 € | / |
